# Association between Regimen Composition and Treatment Response in Patients with Multidrug-Resistant Tuberculosis: A Prospective Cohort Study

**DOI:** 10.1371/journal.pmed.1001932

**Published:** 2015-12-29

**Authors:** Courtney M. Yuen, Ekaterina V. Kurbatova, Thelma Tupasi, Janice Campos Caoili, Martie Van Der Walt, Charlotte Kvasnovsky, Martin Yagui, Jaime Bayona, Carmen Contreras, Vaira Leimane, Julia Ershova, Laura E. Via, HeeJin Kim, Somsak Akksilp, Boris Y. Kazennyy, Grigory V. Volchenkov, Ruwen Jou, Kai Kliiman, Olga V. Demikhova, Irina A. Vasilyeva, Tracy Dalton, J. Peter Cegielski

**Affiliations:** 1 Centers for Disease Control and Prevention, Atlanta, Georgia, United States of America; 2 Tropical Disease Foundation, Manila, Philippines; 3 Medical Research Council, Pretoria, South Africa; 4 National Institute of Health, Lima, Peru; 5 Partners In Health, Boston, Massachusetts, United States of America; 6 Socios en Salud Sucursal, Lima, Peru; 7 Riga East University Hospital Centre of Tuberculosis and Lung Diseases, Riga, Latvia; 8 National Institute of Allergy and Infectious Diseases, National Institutes of Health, Bethesda, Maryland, United States of America; 9 Korean Institute of Tuberculosis, Seoul, Republic of Korea; 10 Department of Disease Control, Ministry of Public Health, Bangkok, Thailand; 11 Orel Oblast Tuberculosis Dispensary, Orel, Russian Federation; 12 Vladimir Oblast Tuberculosis Dispensary, Vladimir, Russian Federation; 13 Taiwan Centers for Disease Control, Taipei, Taiwan; 14 Tartu University Hospital, Tartu, Estonia; 15 Central Tuberculosis Research Institute, Russian Academy of Medical Sciences, Moscow, Russian Federation; McGill University, CANADA

## Abstract

**Background:**

For treating multidrug-resistant tuberculosis (MDR TB), the World Health Organization (WHO) recommends a regimen of at least four second-line drugs that are likely to be effective as well as pyrazinamide. WHO guidelines indicate only marginal benefit for regimens based directly on drug susceptibility testing (DST) results. Recent evidence from isolated cohorts suggests that regimens containing more drugs may be beneficial, and that DST results are predictive of regimen effectiveness. The objective of our study was to gain insight into how regimen design affects treatment response by analyzing the association between time to sputum culture conversion and both the number of potentially effective drugs included in a regimen and the DST results of the drugs in the regimen.

**Methods and Findings:**

We analyzed data from the Preserving Effective Tuberculosis Treatment Study (PETTS), a prospective observational study of 1,659 adults treated for MDR TB during 2005–2010 in nine countries: Estonia, Latvia, Peru, Philippines, Russian Federation, South Africa, South Korea, Thailand, and Taiwan. For all patients, monthly sputum samples were collected, and DST was performed on baseline isolates at the US Centers for Disease Control and Prevention. We included 1,137 patients in our analysis based on their having known baseline DST results for at least fluoroquinolones and second-line injectable drugs, and not having extensively drug-resistant TB. These patients were followed for a median of 20 mo (interquartile range 16–23 mo) after MDR TB treatment initiation. The primary outcome of interest was initial sputum culture conversion. We used Cox proportional hazards regression, stratifying by country to control for setting-associated confounders, and adjusting for the number of drugs to which patients’ baseline isolates were resistant, baseline resistance pattern, previous treatment history, sputum smear result, and extent of disease on chest radiograph.

In multivariable analysis, receiving an average of at least six potentially effective drugs (defined as drugs without a DST result indicating resistance) per day was associated with a 36% greater likelihood of sputum culture conversion than receiving an average of at least five but fewer than six potentially effective drugs per day (adjusted hazard ratio [aHR] 1.36, 95% CI 1.09–1.69). Inclusion of pyrazinamide (aHR 2.00, 95% CI 1.65–2.41) or more drugs to which baseline DST indicated susceptibility (aHR 1.65, 95% CI 1.48–1.84, per drug) in regimens was associated with greater increases in the likelihood of sputum culture conversion than including more drugs to which baseline DST indicated resistance (aHR 1.33, 95% CI 1.18–1.51, per drug). Including in the regimen more drugs for which DST was not performed was beneficial only if a minimum of three effective drugs was present in the regimen (aHR 1.39, 95% CI 1.09–1.76, per drug when three effective drugs present in regimen).

The main limitation of this analysis is that it is based on observational data, not a randomized trial, and drug regimens varied across sites. However, PETTS was a uniquely large and rigorous observational study in terms of both the number of patients enrolled and the standardization of laboratory testing. Other limitations include the assumption of equivalent efficacy across drugs in a category, incomplete data on adherence, and the fact that the analysis considers only initial sputum culture conversion, not reversion or long-term relapse.

**Conclusions:**

MDR TB regimens including more potentially effective drugs than the minimum of five currently recommended by WHO may encourage improved response to treatment in patients with MDR TB. Rapid access to high-quality DST results could facilitate the design of more effective individualized regimens. Randomized controlled trials are necessary to confirm whether individualized regimens with more than five drugs can indeed achieve better cure rates than current recommended regimens.

## Abstract 2

In this prospective cohort study, Courtney Yuen and colleagues examine the association between regimen composition and treatment response in patients with multidrug-resistant tuberculosis.

## Editors' Summary

**Background:**

Tuberculosis (TB)—a contagious bacterial disease that usually infects the lungs—is a global public health problem. The World Health Organization (WHO) estimates that, in 2014, about 9.6 million people developed TB and 1.5 million people died from the disease. *Mycobacterium tuberculosis*, the organism that causes TB, is spread in airborne droplets when people with TB cough. The symptoms of TB include persistent cough, fever, and weight loss; diagnostic tests for TB include the culture (growth) of *M*. *tuberculosis* from sputum samples (mucus coughed up from the lungs) and chest X-rays. TB can be cured by taking several antibiotics (including rifampicin and isoniazid) every day for six months. However, the emergence of multidrug-resistant tuberculosis (MDR TB; TB with resistance to at least both rifampicin and isoniazid) and extensively drug-resistant TB (MDR TB with additional resistance to at least quinolones and to second-line injectable antibiotics) is now threatening TB control efforts—about 5% of the cases of TB that occurred in 2014 were caused by MDR TB.

**Why Was This Study Done?:**

WHO guidelines for the treatment of MDR TB recommend a regimen that includes pyrazinamide and at least four second-line drugs (amikacin or other second-line injectable drugs and fluoroquinolones such as ofloxacin) that are likely to be effective. When bacterial drug susceptibility testing (DST) is not available, determination of the likely effectiveness of drugs is based on patients’ previous exposure to antibiotics and the background drug resistance levels in the community. Although the guidelines state that regimens based on DST results are only marginally more effective than regimens determined without such results, recent evidence suggests that DST results predict regimen effectiveness and that regimens containing more drugs than currently recommended by the guidelines might be beneficial. Here, the researchers undertake a prospective cohort study (an observational study that compares outcomes in non-randomized groups of patients given different treatments) to gain insights into how regimen design affects treatment response among people with MDR TB. Specifically, the researchers analyze the association between both the number of potentially effective drugs included in a regimen and the DST results of the drugs in the regimen and the time to sputum culture conversion (the time between treatment initiation and having two consecutive negative sputum cultures).

**What Did the Researchers Do and Find?:**

The researchers used a statistical approach called multivariable analysis to analyze data (including baseline DST results and information on the time to sputum culture conversion) collected from 1,137 adults with MDR TB who participated in the Preserving Effective Tuberculosis Treatment Study (PETTS). Receiving an average of at least six potentially effective drugs per day (drugs without a baseline DST result indicating resistance) was associated with a 36% greater likelihood of sputum culture conversion than receiving an average of at least five but fewer than six potentially effective drugs per day. Inclusion of more drugs to which baseline DST results indicated susceptibility was associated with a 65% higher likelihood of sputum culture conversion per drug, and inclusion of pyrazinamide was associated with a doubling of the likelihood of sputum culture conversion, Inclusion of more drugs to which baseline DST results indicated resistance was associated with only a 33% higher likelihood of sputum culture conversion per drug. Finally, inclusion of more drugs for which DST had not been performed was beneficial only if the regimen already contained a minimum of three likely effective drugs.

**What Do These Findings Mean?:**

These findings suggest that regimens that include more potentially effective drugs than currently recommended by WHO might improve the response of patients with MDR TB to treatment. In addition, these findings suggest that rapid access to high-quality DST results could facilitate the design of more effective individualized regimens. However, because this study was based on observational data, these findings do not prove that increasing the number of drugs in treatment regimens or basing drug choice on DST results improves outcomes. It is possible that all the patients who received a particular treatment shared an unknown, non-drug-related characteristic that was actually responsible for their response to treatment. Additionally, the authors were not able to assess the effects of individual drugs, and the analysis assumed equivalent efficacy for all drugs in a particular category, which may not be true in reality. The authors also did not have data on adherence or long-term follow-up to assess relapse or sputum culture reversion. Thus, although these findings suggest that changes to the current WHO guidelines for the treatment of MDR TB might be beneficial, randomized controlled trials should be undertaken first to confirm whether individualized regimens that include more than five drugs can achieve better cure rates than the currently recommended regimens.

**Additional Information:**

This list of resources contains links that can be accessed when viewing the PDF on a device or via the online version of the article at http://dx.doi.org/10.1371/journal.pmed.1001932.

- The World Health Organization (WHO) provides information (in several languages) on tuberculosis and on multidrug-resistant tuberculosis (in several languages); its *Guidelines for the Programmatic Management of Drug-Resistant Tuberculosis*: *2011 Update* and the *Global Tuberculosis Report 2015*, which provides up-to-date information about tuberculosis around the world, are available
- The Stop TB Partnership is working towards tuberculosis elimination and provides personal stories about tuberculosis (in English and Spanish)
- The US Centers for Disease Control and Prevention provides information about tuberculosis and about drug-resistant tuberculosis (in English and Spanish), including a brief description of PETTS
- The US National Institute of Allergy and Infectious Diseases also has detailed information on tuberculosis, including a drug-resistant tuberculosis visual tour
- TB & Me, a collaborative blogging project run by patients being treated for MDR TB and Médecins Sans Frontières, provides more patient stories
- MedlinePlus has links to further information about tuberculosis (in English and Spanish)

## Introduction

World Health Organization (WHO) guidelines for the treatment of multidrug-resistant tuberculosis (MDR TB) recommend a regimen consisting of at least four second-line drugs that are likely to be effective as well as pyrazinamide [1]. In the absence of drug susceptibility testing (DST) results for a patient’s isolate, likely effectiveness is determined based on previous exposure to a drug, background resistance levels to that drug in the community, and, in patients who were contacts to other known cases, DST results for an associated case. Furthermore, the guidelines indicate that only marginal benefit has been observed for regimens based directly on the DST results for a patient’s isolate [1].

A meta-analysis of cohort studies of patients with MDR TB reported that in vitro susceptibility to individual drugs was consistently and statistically significantly associated with higher odds of treatment success compared to in vitro resistance, suggesting clinical utility for DST in regimen design [2]. In addition, the use of baseline DST results to design individualized regimens involving prolonged use of five or more drugs with likely effectiveness has been associated with decreased risks of treatment failure, death, and relapse among patient cohorts in Peru and the Russian Federation [3–5]. Together, this evidence suggests the need to reassess both the role of DST in regimen design as well as the potential benefit of including more drugs in MDR TB regimens.

To gain insight into how regimen design affects treatment response, we analyzed treatment and microbiological data from the Preserving Effective Tuberculosis Treatment Study (PETTS), a 6-y, multinational prospective cohort study of patients with MDR TB [6]. As our goal was to focus on the association between DST results and the direct microbiological effect of drugs, we used time to sputum culture conversion as an indicator of the bactericidal effect of treatment. We assessed the association between the number of potentially effective drugs included in a regimen and time to sputum culture conversion. In addition, we compared the individual effects of drugs to which DST results indicated susceptibility, drugs to which DST results indicated resistance, and drugs that were not tested.

## Methods

### Ethics

PETTS was approved by the US Centers for Disease Control and Prevention (CDC) Institutional Review Board and institutional review boards at all participating sites. Written informed consent was obtained from all study participants.

### Patient Population and Study Procedures

The PETTS study design and patient population have been described previously [6]. Briefly, this prospective cohort study, conducted in 2005–2010, enrolled consecutive adults with pulmonary MDR TB in nine countries: Estonia (nationwide), Latvia (nationwide), Peru (two districts in Lima), Philippines (greater Manila), Russian Federation (Orel and Vladimir Oblasts), South Africa (Eastern Cape, KwaZulu-Natal, Mpumalanga, and Northwest provinces), South Korea (National Masan Tuberculosis Hospital, Masan, and Korean Institute of Tuberculosis, Seoul), Thailand (Sakon Nakon, Srisaket, Ubon Ratchathani, and Yasothon provinces), and Taiwan (nationwide). Inclusion criteria for the study were (1) pulmonary MDR TB confirmed microbiologically by a local reference laboratory from a specimen collected within 30 d of starting treatment and (2) receipt of second-line drugs for at least 30 d. South Africa restricted enrollment to patients who had not previously been treated for MDR TB. Standardized information was recorded at all sites, including demographic, socioeconomic, and clinical information for each participant, and treatment and laboratory monitoring details.

Culture was performed on a baseline sputum sample, and monthly follow-up sputum samples were collected for the duration of treatment. Local laboratories performed cultures for monitoring and DST for determining patient eligibility. A subset of isolates from patients enrolled in the study were shipped in batches to CDC for centralized DST and genotyping. Patients were eligible for inclusion in this analysis if they had positive cultures at the start of treatment for MDR TB, if they had DST results from CDC for fluoroquinolones (DST was performed for ciprofloxacin and ofloxacin) and second-line injectable drugs (i.e., amikacin, kanamycin, capreomycin), and if resistance to both isoniazid and rifampin were confirmed at CDC. We excluded patients for whom the DST performed at CDC indicated susceptibility to either isoniazid or rifampin in response to a reviewer suggestion, as several of these patients were treated with isoniazid or rifampin. Patients with extensively drug-resistant tuberculosis (XDR TB), defined as MDR TB with additional resistance to any fluoroquinolone and at least one second-line injectable drug, were excluded from the analysis, as were patients for whom a date of culture conversion or censoring could not be determined.

The primary research objective of PETTS was to determine whether the Green Light Committee approval process was associated with reduced amplification of drug resistance; the results of this analysis have been previously reported [7]. However, the study protocol was conceived to produce a dataset that could be used to answer several additional research questions that required rigorous microbiological follow-up of MDR TB patients. The present analysis was not contained in the original analysis plan, but was conceived because several recent publications suggested that regimens based on known drug susceptibilities and regimens containing more drugs were associated with better clinical outcomes [2–5].

### Definitions

Initial sputum culture conversion was defined as at least two consecutive negative cultures of sputum samples collected at least 30 d apart. Time to sputum culture conversion was defined as the time in days from the start of MDR TB treatment to the sputum specimen collection date of the first of the consecutive negative cultures. Patients for whom sputum culture conversion did not occur were censored 1 mo before the collection date of the last sputum specimen because they were still at risk to convert during the last month of follow-up.

Classification of each drug’s effectiveness was based on the results of DST performed at CDC on the baseline culture using the indirect agar plate proportion method [6]. DST was performed for isoniazid, rifampin, ethambutol, ciprofloxacin, ofloxacin, amikacin, capreomycin, kanamycin, streptomycin, rifabutin, ethionamide, and para-aminosalicylic acid. Drugs for which DST indicated susceptibility were considered effective. Drugs for which the baseline DST result indicated resistance were considered ineffective. In addition, levofloxacin and moxifloxacin were considered effective if no resistance to ciprofloxacin or ofloxacin was observed, and were considered ineffective if resistance to either ciprofloxacin or ofloxacin was observed. Prothionamide was considered effective if no resistance to ethionamide was observed, and ineffective if resistance to ethionamide was observed. Drugs for which DST was not performed at CDC (cycloserine, terizidone, amoxicillin/clavulanate, clarithromycin, thioacetazone, clofazimine, imipenem, and linezolid) were classified as untested drugs. Pyrazinamide, although not tested routinely, was kept separate from this group of untested drugs because it is a first-line drug with a well-established role in treatment, and it is recommended for routine inclusion in MDR TB regimens [1].

For each individual drug, we calculated the number of days during which the drug was included in a patient’s regimen between initiation of MDR TB treatment and sputum culture conversion or censoring. The number of days a drug was included in a patient’s regimen was inferred from the dates the drug was started and stopped; if a single drug was started and stopped multiple times, the days between each pair of start and stop dates were summed. Drug-days were summed for all the drugs in each of four groups: effective drugs, ineffective drugs, pyrazinamide, and untested drugs. For each group, this sum was divided by the number of days before sputum culture conversion or censoring to calculate the average number of drugs in each group that the patient received per day. In addition, a composite variable was created to reflect the total number of “potentially effective drugs” received per day, which included all effective drugs, pyrazinamide, and untested drugs.

### Data Analysis

We analyzed the association between variables of interest and sputum culture conversion using Cox proportional hazards regression. We stratified by country to control for setting-associated confounders. We evaluated proportional hazards assumptions by testing the significance of time-dependent interaction terms for all variables. We were interested in the associations between time to sputum culture conversion and both the number of drugs in a regimen and the presumed effectiveness of these drugs. Therefore, we generated two multivariable models to assess the association between treatment regimen and time to sputum culture conversion. In the first model, the exposure of interest was the average number of potentially effective drugs received per day, analyzed as a categorical variable. In the second model, the exposures of interest were the average numbers of drugs received in each of the four drug groups, analyzed as continuous variables. We considered clinical and demographic covariates for inclusion in the multivariable models based on the strength of univariate associations with sputum culture conversion (covariates with Wald *p* < 0.1 were eligible for inclusion) or biological plausibility. The resistance pattern at baseline and the number of drugs to which the baseline isolate was resistant were retained in both models because of an established association between the extent of baseline drug resistance and treatment success [8] and because the extent of drug resistance was likely to be associated with resistance to untested drugs. To generate the final models, we used backward elimination, assessing the effect of each elimination on the point estimates and confidence intervals to identify potential confounders.

In the second model, we believed interactions among the different drug groups to be likely. Therefore, we assessed both the main effects model and a model in which we considered all two-way interactions among drug group variables. Collinearity among variables was assessed; a variance inflation factor > 5 or a maximum condition index > 50 were considered evidence of collinearity. As a sensitivity analysis, we restricted the first model to patients who did not receive any Group 4 (oral second-line drugs other than fluoroquinolones) or Group 5 drugs (drugs with antimycobacterial activity but unproven efficacy against drug-resistant TB) [1] for which drug sensitivity was unknown. All analyses were performed using SAS 9.3.

## Results

**Fig 1:**
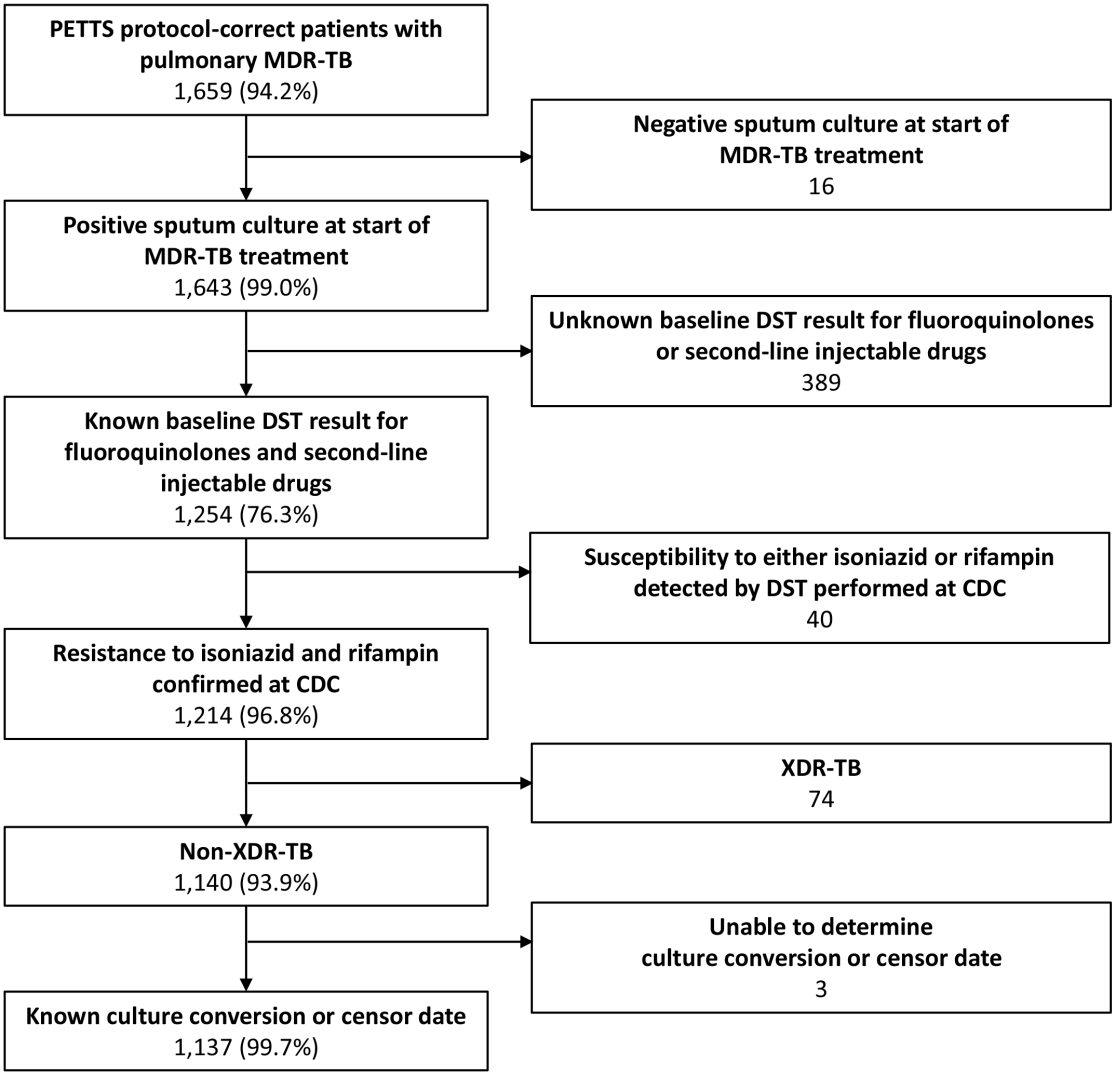
Inclusion of patients in the analysis. MDR-TB is tuberculosis resistant to at least isoniazid and rifampin; XDR-TB is tuberculosis resistant to at least isoniazid, rifampin, one fluoroquinolone, and one second-line injectable drug.

Out of 1,659 patients in the PETTS cohort, 1,137 were included in our analysis (Fig 1). These patients were followed for a median of 20 mo (interquartile range 16–23 mo) after MDR TB treatment initiation. Initial sputum culture conversion occurred for 909 (79.9%) patients at a median of 2 mo (interquartile range 1–3 mo). However, the percentage of patients achieving initial sputum culture conversion within 6 mo varied considerably by country (Table 1).

**Table 1:** Baseline drug resistance and treatment characteristics of patients, by country (*n =* 1,137).

Characteristic	Country								
	Estonia	Latvia	Peru	Philippines	Russian Federation	South Africa	South Korea	Taiwan	Thailand
**Number of patients included in analysis**	22	80	162	374	86	252	75	38	48
**Site approved by Green Light Committee**	Yes	Yes	Yes	Yes	Yes	No	No	No	No
**Number of drugs to which TB resistant at baseline** *	5 (3–8)	5 (2–10)	4 (2–9)	5 (2–8)	5 (2–9)	4 (2–10)	4 (2–9)	3 (2–7)	4 (2–8)
**Resistance pattern at baseline** ^†^									
MDR only	13 (59%)	41 (51%)	135 (83%)	348 (93%)	56 (65%)	195 (77%)	53 (71%)	30 (79%)	43 (90%)
MDR with resistance to any second-line injectable	6 (27%)	34 (43%)	22 (14%)	5 (1%)	22 (26%)	51 (20%)	7 (9%)	1 (3%)	2 (4%)
MDR with resistance to any fluoroquinolone	3 (14%)	5 (6%)	5 (3%)	21 (6%)	8 (9%)	6 (2%)	15 (20%)	7 (18%)	3 (6%)
**Previous treatment history** ^†^									
None	16 (73%)	45 (56%)	24 (15%)	0 (0%)	31 (36%)	9 (4%)	9 (12%)	22 (58%)	2 (4%)
First-line drugs only	2 (9%)	20 (25%)	111 (69%)	330 (88%)	33 (38%)	235 (93%)	28 (37%)	15 (39%)	42 (88%)
Second-line drugs	4 (18%)	14 (18%)	18 (11%)	44 (12%)	18 (21%)	8 (3%)	33 (44%)	1 (3%)	4 (8%)
Unknown	0 (0%)	1 (1%)	9 (6%)	0 (0%)	4 (5%)	0 (0%)	5 (7%)	0 (0%)	0 (0%)
**Average number of potentially effective drugs received per day** ^†^									
0 to <4	1 (5%)	13 (16%)	4 (2%)	76 (20%)	29 (34%)	102 (40%)	17 (23%)	6 (16%)	16 (33%)
4 to <5	4 (18%)	27 (34%)	12 (7%)	144 (39%)	35 (41%)	86 (34%)	22 (29%)	12 (32%)	18 (38%)
5 to <6	10 (45%)	29 (36%)	47 (29%)	130 (35%)	22 (26%)	55 (22%)	21 (28%)	15 (39%)	13 (27%)
6 or more	7 (32%)	11 (14%)	99 (61%)	24 (6%)	0 (0%)	9 (4%)	15 (20%)	5 (13%)	1 (2%)
**Patients with initial sputum culture conversion within 6 mo, by average number of potentially effective drugs received** ^‡^									
0 to <4	0 (0%)	7 (54%)	2 (50%)	70 (92%)	22 (76%)	21 (21%)	6 (35%)	5 (83%)	11 (69%)
4 to <5	1 (25%)	24 (89%)	4 (33%)	138 (96%)	26 (74%)	44 (51%)	7 (32%)	10 (83%)	12 (67%)
5 to <6	6 (60%)	27 (93%)	28 (59%)	128 (98%)	19 (86%)	44 (80%)	12 (57%)	15 (100%)	13 (100%)
6 or more	7 (100%)	7 (64%)	81 (82%)	23 (96%)	N/A	9 (100%)	12 (80%)	5 (100%)	1 (100%)
**Patients with initial sputum culture conversion within 12 mo, by average number of potentially effective drugs received** ^‡^									
0 to <4	0 (0%)	7 (54%)	2 (50%)	71 (93%)	23 (79%)	28 (27%)	7 (41%)	5 (83%)	13 (81%)
4 to <5	1 (25%)	24 (89%)	7 (58%)	139 (97%)	29 (83%)	50 (58%)	8 (36%)	11 (92%)	13 (72%)
5 to <6	6 (60%)	27 (93%)	32 (68%)	128 (98%)	19 (86%)	48 (87%)	12 (57%)	15 (100%)	13 (100%)
6 or more	7 (100%)	7 (64%)	86 (87%)	23 (96%)	N/A	9 (100%)	12 (80%)	5 (100%)	1 (100%)

Initial sputum culture conversion was defined as at least two consecutive negative cultures of sputum samples collected at least 30 d apart.

*Median (range) presented.

^†^Number (column percent) presented.

^‡^Number (percent of those who received that number of drugs) presented.

MDR, multidrug resistant; N/A, not applicable.

**Fig 2:**
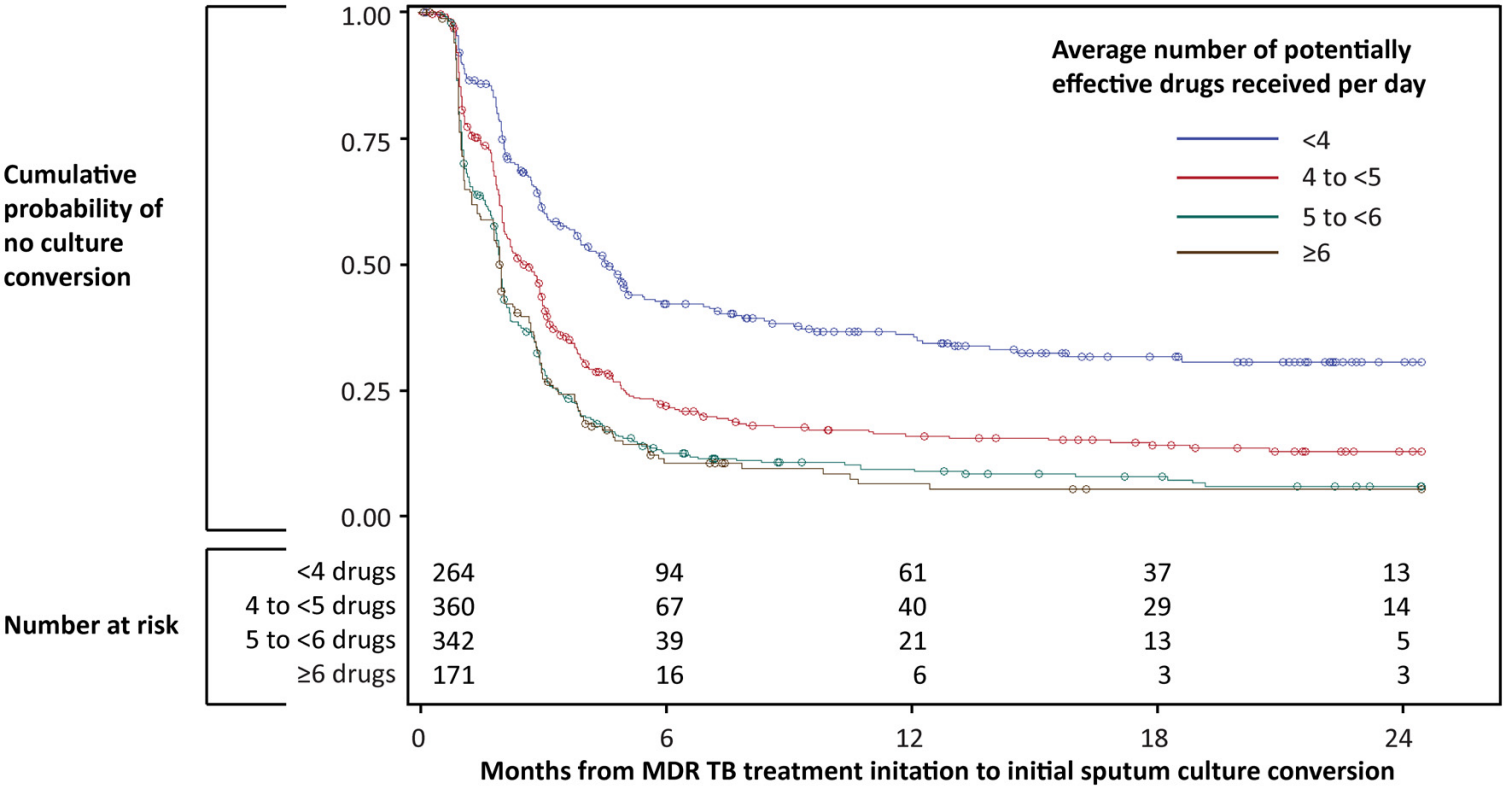
Time to initial sputum culture conversion by average number of potentially effective drugs received per day. Initial sputum culture conversion was defined as at least two consecutive negative cultures of sputum samples collected at least 30 d apart.

Time to initial sputum culture conversion among all patients by average number of potentially effective drugs received per day is show graphically in Fig 2. As baseline drug resistance pattern, drug exposure, and percentage of patients achieving initial sputum culture conversion by 6 mo varied by country (Table 1), we stratified the statistical analysis by country to control for setting-associated confounders. In stratified univariate analysis, receiving an average of at least six potentially effective drugs per day was associated with a 34% increase in the likelihood of sputum culture conversion compared to receiving an average of at least five but fewer than six potentially effective drugs per day (hazard ratio [HR] 1.34 per effective drug, 95% CI 1.08–1.65) (Table 2). In contrast, receiving fewer potentially effective drugs was associated with lower likelihoods of sputum culture conversion (HR for fewer than four drugs 0.37, 95% CI 0.30–0.45; HR for at least four but fewer than five drugs 0.58, 95% CI 0.49–0.68).

**Table 2:** Patient characteristics and univariate associations with sputum culture conversion among patients treated for MDR TB (*n =* 1,137).

Predictor	*n* (Percent) or Median (Range)	HR (95% CI)
**Average number of potentially effective drugs received per day**		
0 to <4	264 (23%)	**0.37 (0.30–0.45**)
4 to <5	360 (32%)	**0.58 (0.49–0.68)**
5 to <6	342 (30%)	Reference
6 or more	171 (15%)	**1.34 (1.08–1.65)**
**Average number of effective drugs received per day**	3 (0–6.0)	**1.45 (1.34–1.56) per drug**
**Average number of ineffective drugs received per day**	0.6 (0–4.5)	0.94 (0.86–1.03) per drug
**Average doses of pyrazinamide received per day**	1 (0–1.0)	**1.94 (1.62–2.32) per dose**
**Average number of untested drugs received per day**	1 (0–4.0)	**0.84 (0.73–0.96) per drug**
**Number of drugs to which TB resistant at baseline**	4 (2–10)	**0.87 (0.83–0.92) per drug**
**Resistance pattern at baseline**		
MDR only	914 (80%)	Reference
MDR with resistance to any second-line injectable	150 (13%)	**0.58 (0.45–0.74)**
MDR with resistance to any fluoroquinolone	73 (6%)	**0.44 (0.32–0.60)**
**Hospitalized at enrollment**		
No	617 (54%)	Reference
Yes	520 (45%)	**0.68 (0.51–0.91)**
**Previous treatment history**		
None	158 (14%)	Reference
First-line drugs only	816 (72%)	0.84 (0.66–1.06)
Second-line drugs	144 (13%)	**0.56 (0.42–0.74)**
Unknown	19 (2%)	**0.49 (0.26–0.93)**
**Smear result**		
Negative	102 (9%)	Reference
Positive	999 (88%)	**0.73 (0.57–0.94)**
Unknown	36 (3%)	**0.22 (0.11–0.44)**
**Extent of disease on chest radiograph**		
Unilateral	215 (19%)	Reference
Bilateral	901 (79%)	**0.72 (0.61–0.85)**
Unknown	21 (2%)	1.50 (0.93–2.42)
**Site of disease**		
Pulmonary only	1,087 (96%)	Reference
Pulmonary and extrapulmonary	49 (4%)	0.96 (0.70–1.32)
**Evidence of cavity on chest radiograph**		
No	424 (37%)	Reference
Yes	690 (61%)	**0.83 (0.72–0.95)**
Unknown	23 (2%)	1.20 (0.75–1.92)
**HIV status**		
Negative	542 (48%)	Reference
Positive	145 (13%)	0.94 (0.71–1.26)
Unknown	450 (40%)	**1.55 (1.10–2.17)**
**Diabetes mellitus**		
No	978 (86%)	Reference
Yes	154 (14%)	1.02 (0.85–1.24)
**Age (in years)**	36 (18–81)	0.95 (0.90–1.01) per 10 y
**Alcohol abuse**		
No	928 (82%)	Reference
Yes	167 (15%)	0.85 (0.68–1.07)
Unknown	43 (4%)	**0.59 (0.35–0.99)**
**Smokes tobacco**		
No	872 (77%)	Reference
Yes	252 (22%)	0.83 (0.65–1.04)
Unknown	13 (1%)	0.71 (0.29–1.71)

HRs result from univariate Cox proportional hazards regression analysis for each variable, stratified by country, with time to initial sputum culture conversion as the outcome. Initial sputum culture conversion was defined as at least two consecutive negative cultures of sputum samples collected at least 30 d apart. HRs in bold are statistically significant.

MDR, multidrug resistant.

In univariate analysis, stratified by country, the presence of more effective drugs in the regimen was associated with an increased likelihood of sputum culture conversion (HR 1.45 per effective drug, 95% CI 1.34–1.56), and inclusion of pyrazinamide in the regimen was associated with a doubled likelihood of sputum culture conversion (HR 1.94, 95% CI 1.62–2.32) (Table 2). In contrast, the presence of more untested drugs in the regimen was associated with a slightly but significantly decreased likelihood of sputum culture conversion (HR 0.84 per untested drug, 95% CI 0.73–0.96). The following were all associated with a lower likelihood of sputum culture conversion: baseline resistance to more drugs (HR 0.87, 95% CI 0.83–0.92, per drug), baseline resistance specifically to fluoroquinolones (HR 0.44, 95% CI 0.32–0.60) or second-line injectable drugs (HR 0.58, 95% CI 0.45–0.74), hospitalization at enrollment (HR 0.68, 95% CI 0.51–0.91), previous treatment with second-line drugs (HR 0.56, 95% CI 0.42–0.74), positive baseline sputum smear microscopy result (HR 0.73, 95% CI 0.57–0.94), radiographically determined bilateral disease (HR 0.72, 95% CI 0.61–0.85), and evidence of cavity formation (HR 0.83, 95% CI 0.72–0.95) (Table 2).

The results of the multivariable analyses are summarized in Table 3. In the first multivariable model, receiving an average of at least six potentially effective drugs per day was associated with a 36% greater likelihood of sputum culture conversion than receiving an average of at least five but fewer than six potentially effective drugs per day (adjusted hazard ratio [aHR] 1.36, 95% CI 1.09–1.69), after adjusting for extent and pattern of baseline resistance, previous treatment history, sputum smear result, and extent of disease on chest radiograph. In contrast, receiving fewer potentially effective drugs was associated with lower likelihoods of sputum culture conversion (aHR for fewer than four drugs 0.36, 95% CI 0.29–0.44; aHR for at least four but fewer than five drugs 0.56, 95% CI 0.47–0.66). Similar results were observed when we excluded from analysis 697 patients who received any Group 4 or 5 drug for which drug susceptibility was unknown (S1 Table).

**Table 3:** Multivariable models for the association between regimen composition and sputum culture conversion.

Predictor	aHR (95% CI)	
	Model 1	Model 2
**Average number of potentially effective drugs received per day**		Not included in model
0 to <4	**0.36 (0.29–0.44)**	
4 to <5	**0.56 (0.47–0.66)**	
5 to <6	Reference	
6 or more	**1.36 (1.09–1.69)**	
**Average number of effective drugs received per day** *	Not included in model	**1.65 (1.48–1.84) per drug, when regimen contains one untested drug**
**Average number of ineffective drugs received per day** *	Not included in model	**1.33 (1.18–1.51) per drug**
**Average doses of pyrazinamide received per day** *	Not included in model	**2.00 (1.65–2.41) per drug, when regimen contains one untested drug**
**Average number of untested drugs received per day** *	Not included in model	See Table 4
**Number of drugs to which TB resistant at baseline** *	**1.09 (1.02–1.17)**	1.07 (0.99–1.16) per drug
**Resistance pattern at baseline**		
MDR only	Reference	Reference
MDR with resistance to any second-line injectable	**0.51 (0.38–0.70)**	**0.56 (0.42–0.76)**
MDR with resistance to any fluoroquinolone	**0.52 (0.36–0.73)**	**0.48 (0.34–0.68)**
**Previous treatment history**		
None	Reference	Reference
First-line drugs only	0.84 (0.66–1.06)	0.84 (0.67–1.07)
Second-line drugs	**0.62 (0.46–0.83)**	**0.72 (0.54–0.97)**
Unknown	**0.46 (0.24–0.87)**	0.53 (0.28–1.02)
**Smear result**		
Negative	Reference	Reference
Positive	**0.72 (0.56–0.92)**	**0.73 (0.56–0.94)**
Unknown	**0.25 (0.12–0.50)**	**0.22 (0.10–0.46)**
**Extent of disease on chest radiograph**		
Unilateral	Reference	Reference
Bilateral	**0.70 (0.59–0.83)**	**0.68 (0.57–0.80)**
Unknown	1.16 (0.71–1.87)	1.09 (0.67–1.78)

aHRs result from two separate Cox proportional hazards regression models for the association between regimen composition and time to initial sputum culture conversion. All variables included in each model are shown, and analysis was stratified by country. Model 1 characterizes regimens using the average number of potentially effective drugs received, treated as a categorical variable. Model 2 characterizes regimens using the average number of drugs in each of four categories, treated as continuous variables. Model 2 included the following two interaction terms: average number of effective drugs received per day × average number of untested drugs received per day; average doses of pyrazinamide received per day × average number of untested drugs received per day. Initial sputum culture conversion was defined as at least two consecutive negative cultures of sputum samples collected at least 30 d apart. aHRs in bold are statistically significant.

*Continuous variables.

MDR, multidrug resistant.

In the second multivariable model, HRs associated with each drug group exposure of interest were adjusted for the other drug group exposures, as well as for extent and pattern of baseline resistance, previous treatment history, sputum smear result, and extent of disease on chest radiograph. The main effects model produced similar estimates as the model that considered interaction terms; therefore, we present the model that included the interaction terms, as significant interactions were detected among effective drugs, untested drugs, and pyrazinamide. For patients receiving one untested drug (the median number of untested drugs received), the presence of one additional effective drug in the regimen was associated with a 65% greater likelihood of sputum culture conversion (aHR 1.65, 95% CI 1.48–1.84), and including pyrazinamide in the regimen was associated with a doubled likelihood of sputum culture conversion (aHR 2.00, 95% CI 1.65–2.41). The presence of an additional ineffective drug in a regimen was associated with a 33% greater likelihood of sputum culture conversion (aHR 1.33, 95% CI 1.18–1.51), despite resistance to ineffective drugs on baseline DST (Table 3).

The benefit of including untested drugs was dependent on the other drugs present in the regimen (Table 4). For patients receiving two or fewer effective drugs, the presence of an additional untested drug was not associated with any significant acceleration of sputum culture conversion. For patients receiving three effective drugs (the median number of effective drugs received) and no pyrazinamide, the presence of an additional untested drug was associated with a 39% greater likelihood of sputum culture conversion (aHR 1.39, 95% CI 1.09–1.76). However, for patients receiving three effective drugs as well as pyrazinamide, the presence of an additional untested drug was not associated with any significant increase in the likelihood of sputum culture conversion (aHR 0.93, 95% CI 0.77–1.11). Additional results relating to these interactions are included in S2 and S3 Tables. Drug exposures included in the untested drug group were predominantly cycloserine and terizidone (47% and 25% of drug-days of exposure, respectively), with Group 5 drugs comprising the remainder (a cumulative 28% of drug-days of exposure). Neither bedaquiline nor delamanid, nor any experimental drugs, were used in this cohort.

**Table 4:** Multivariable model 2 adjusted hazard ratios for sputum culture conversion associated with inclusion of one additional untested drug in the regimen, stratified by average number of effective drugs received per day and inclusion of pyrazinamide in the regimen.

Average Number of Effective Drugs Received per Day*	Pyrazinamide Included in Regimen	
	No	Yes
**0**	0.78 (0.51–1.17)	0.52 (0.34–0.79)
**1**	0.94 (0.69–1.29)	0.63 (0.46–0.86)
**2**	1.14 (0.89–1.47)	0.76 (0.61–0.96)
**3**	**1.39 (1.09–1.76)**	0.93 (0.77–1.11)
**4**	**1.68 (1.27–2.23)**	1.13 (0.91–1.40)
**5**	**2.04 (1.42–2.94)**	**1.37 (1.01–1.85)**
**6**	**2.48 (1.56–3.95)**	**1.66 (1.10–2.49)**

Data presented are aHRs (95% CIs). aHRs are adjusted for average number of ineffective drugs received per day, average number of untested drugs received per day, extent and pattern of baseline resistance, previous treatment history, sputum smear result, and extent of disease. Analysis was stratified by country. Initial sputum culture conversion was defined as at least two consecutive negative cultures of sputum samples collected at least 30 days apart. 95% confidence intervals given in parentheses. aHRs in bold are statistically significant.

*Median: 3; range: 0–6.

Independent associations between clinical covariates and sputum culture conversion were similar in both multivariable models (Table 3). Compared to MDR TB with susceptibility to both second-line injectable drugs and fluoroquinolones, MDR TB with added resistance to any second-line injectable drug (model 1, aHR 0.51, 95% CI 0.38–0.70; model 2, aHR 0.56, 95% CI 0.42–0.76) and MDR TB with added resistance to any fluoroquinolone (model 1, aHR 0.52, 95% CI 0.36–0.73; model 2, aHR 0.48, 95% CI 0.34–0.68) were associated with a lower likelihood of sputum culture conversion. The following were independently associated with a decreased likelihood of sputum culture conversion: prior treatment with second-line tuberculosis drugs (model 1, aHR 0.62, 95% CI 0.46–0.83; model 2, aHR 0.72, 95% CI 0.54–0.97), positive sputum smear (model 1, aHR 0.72, 95% CI 0.56–0.92; model 2, aHR 0.73, 95% CI 0.56–0.94), and bilateral disease (model 1, aHR 0.70, 95% CI 0.59–0.83; model 2, aHR 0.68, 95% CI 0.57–0.80). No collinearity was observed among variables included in the multivariable models.

## Discussion

In our analysis, greater numbers of potentially effective drugs in an MDR TB treatment regimen were associated with accelerated sputum culture conversion. In general, inclusion of pyrazinamide or additional drugs to which baseline DST indicated susceptibility (i.e., effective drugs) were associated with greater increases in the likelihood of sputum culture conversion than inclusion of drugs to which baseline DST indicated resistance (i.e., ineffective drugs). The presence of untested drugs in the regimen was associated with an increased likelihood of sputum culture conversion only if a minimum number of effective drugs was present in the regimen.

We observed a benefit to receiving a greater number of potentially effective drugs (i.e., drugs without a DST result indicating resistance), as well as an interaction in which the presence of more effective drugs enhanced the benefit of untested drugs. Both of these results add to existing evidence that increasing the number of drugs in MDR TB regimens is advantageous. Patients receiving individualized regimens containing a minimum of five probably effective drugs for prolonged periods after sputum culture conversion have been shown to have decreased risks of treatment failure, death, and relapse compared to patients receiving fewer drugs [3–5]. In addition, high cure rates have been reported with only 9 mo of treatment using a standardized regimen including seven drugs during the intensive phase [9]. In response to the accumulating evidence for the benefit of increasing the number of drugs in regimens for MDR TB, WHO guidelines for MDR TB regimen composition increased the minimum number of drugs recommended from four in 2006 and 2008 to five in 2011 [1,10]. Our results suggest that treatment might be further fortified by adding additional potentially effective drugs.

In our analysis, for most patients, the increased likelihood of sputum culture conversion associated with the presence of an additional effective drug in the regimen was greater than the acceleration associated with the presence of an additional ineffective drug. This result suggests that tailoring regimens based on DST results could improve treatment response. However, we also observed a strong benefit to inclusion of pyrazinamide despite unknown efficacy, which would appear to support the WHO recommendation to include pyrazinamide in MDR TB regimens [1] without relying on DST results, which are known to be unreliable for this drug [11]. But as there were no DST results for pyrazinamide in this cohort, caution must be taken when interpreting this finding. It is possible that clinicians accurately assessed the likelihood that pyrazinamide would be effective before deciding to use it, that the prevalence of pyrazinamide resistance in the analyzed cohort was relatively low, or that the efficacy of pyrazinamide in those patients with pyrazinamide susceptibility was so great that an association was observed even though the drug was ineffective in a proportion of the patients who received it. In situations where undetected resistance renders pyrazinamide ineffective, a five-drug regimen that includes pyrazinamide would actually contain only four effective drugs, which could put patients at risk for poorer outcomes [12].

The drugs classified in our analysis as untested drugs comprise those classified as Group 5 by WHO [10], as well as the second-line drugs cycloserine and terizidone. Cycloserine was not tested at CDC because testing requires Lowenstein–Jensen medium, and CDC performs DST using Middlebrook medium or BACTEC broth medium. Because of inconclusive evidence about the efficacy of Group 5 drugs, WHO recommends that they be used only to supplement regimens if additional drugs are required [1]. Consistent with their supplementary role, we observed that the benefit of including untested drugs was dependent on the other drugs present in the regimen. Without a minimum number of effective drugs present, inclusion of an additional untested drug was not associated with any significant increase in the likelihood of sputum culture conversion. Thus, our results suggest that it would be preferential to use the new drugs bedaquiline and delamanid—which are almost certainly likely to be effective because of their novel mechanisms and lack of prior use—in place of Group 5 drugs in treatment regimens.

Unexpectedly, we observed that the addition of ineffective drugs was associated with a modest but significant acceleration of sputum culture conversion. Possible explanations for this observation include infection with multiple strains with differing drug susceptibilities, strains with low-level resistance in vitro that were still susceptible in vivo to therapeutic drug concentrations, or synergistic effects between drugs. It is also possible that misclassification of drug resistance occurred from our using ciprofloxacin and ofloxacin DST results to determine the likely efficacy of moxifloxacin, as moxifloxacin susceptibility has been reported in approximately 30% of clinical isolates resistant to ofloxacin [13,14] or ciprofloxacin [14]. However, in our study population, only 31 (2.7%) patients received moxifloxacin and had it classified as ineffective based on resistance to ofloxacin or ciprofloxacin; therefore, the influence of this misclassification on our results as a whole was likely limited.

The associations with clinical covariates that we observed are consistent with previous studies involving patients with MDR TB that have shown associations between decreased likelihood of sputum culture conversion and sputum smear positivity [15–17], bilateral extent of disease [18], previous treatment with second-line drugs [16,18], and more extensive baseline drug resistance [16,18].

Our analysis was subject to several important limitations. PETTS was an observational study, not a randomized trial. In addition, while the study involved sites in nine different countries, our results may not be generalizable to other settings with very different MDR TB epidemics. For instance, the prevalence of pre–XDR TB fluoroquinolone resistance in the PETTS cohort was substantially lower than has been reported among MDR TB patients in some South Asian settings [19,20]. Choice of regimen varied across sites, as not all countries had all drugs available. While we attempted to reduce bias by stratifying analysis by country and including clinical covariates in our analysis, complete elimination of bias in a cohort of this diversity is impossible. However, despite the limitations inherent to using observational data from treatment programs, conclusions drawn from these data may in fact be more easily translatable to clinical decision-making since they reflect results from ordinary practice.

A second set of limitations relates to assumptions made about drug efficacy. The range of baseline resistance patterns and regimens received prevented us from assessing the effects of individual drugs and from controlling for the varying efficacy of individual drugs. Thus, in our analysis, the calculation of the average number of drugs received per day in a particular category gave equal weight to all the drugs in that category even though, in reality, some drugs may exert more of an effect than others. Furthermore, as we considered only baseline DST results when determining drug effectiveness, our analysis did not take into account changing efficacy resulting from acquisition of drug resistance during treatment. However, we believe that relatively few patients in our analytic cohort are likely to have acquired resistance prior to sputum culture conversion or censoring, as only 68 PETTS patients acquired XDR TB during the course of the study [7].

Our analysis was also limited by the data collected. Our quantification of drug exposure was derived from the start and stop dates for individual drugs. However, the PETTS protocol specified that a stoppage need be recorded only if a drug was discontinued for at least 2 wk. Therefore, our measures of drug exposure may be overestimates in some cases because brief interruptions lasting less than 2 wk might not have been recorded. In addition, DST was not performed for every drug that patients received, which prevented us from determining the likely efficacy of all drugs included in the analysis. We also did not have adherence data corresponding to each episode of drug prescription, and we assumed that prescription of a drug was equivalent to its having been taken. This assumption was likely valid for Green Light Committee–approved programs, in which all treatment was administered under direct observation. If adherence was poorer in countries without a Green Light Committee–approved program, we would expect associations between drug exposure and sputum culture conversion to be attenuated, biasing our results toward the null.

Finally, the interpretation of our results is limited by our use of time to initial sputum culture conversion as the outcome. We did not have long-term follow-up data to assess the risk of relapse, nor did we consider sputum culture reversion (i.e., positive cultures obtained after initial sputum culture conversion) in our analysis. Time to initial sputum culture conversion is a standard marker for treatment response in patients with tuberculosis, and has been shown to correlate with treatment outcome in patients with MDR TB [18]. In the PETTS cohort, sputum culture conversion at 6 mo was found to be a robust predictor of final treatment outcome [21]. However, our analysis could have been strengthened had we been able to assess the risk of relapse as well, thereby allowing a comparison between the early prognostic marker of initial sputum culture conversion and long-term treatment efficacy.

In conclusion, our analysis suggests that MDR TB regimens including more potentially effective drugs than the minimum of five currently recommended by WHO may encourage improved response to treatment in patients with MDR TB. In addition, rapid access to high-quality DST results could facilitate the design of more effective individualized regimens. However, randomized controlled trials are necessary to confirm whether individualized regimens with more than five drugs can indeed achieve better cure rates than current recommended regimens, and new rapid DST techniques will be required if knowledge of baseline drug resistance is to guide regimen composition at the beginning of treatment.

## Supporting Information

S1 Table Model 1: sensitivity analysis. (PDF)

S2 Table Model 2: interaction between effective drugs and untested drugs. (PDF)

S3 Table Model 2: interaction between pyrazinamide and untested drugs. (PDF)

S1 Text STROBE statement. Checklist of items that should be included in reports of observational studies.
(PDF)

S2 Text PETTS protocol primary analysis plan. Original plan for primary analysis that was submitted as part of the institutional-review-board-approved PETTS protocol.
(PDF)

S3 Text PETTS preliminary secondary analysis plan. Earliest articulation of planned secondary analyses; secondary analyses were understood by all investigators to be dynamic and not limited to those identified in this presentation.
(PDF)

## Abbreviations

aHR: adjusted hazard ratio
CDC: US Centers for Disease Control and Prevention
DST: drug susceptibility testing
HR: hazard ratio
MDR TB: multidrug-resistant tuberculosis
PETTS: Preserving Effective Tuberculosis Treatment Study
WHO: World Health Organization
XDR TB: extensively drug-resistant tuberculosis
