# Supplementary material for: Association between Regimen Composition and Treatment Response in Patients with Multidrug-Resistant Tuberculosis: A Prospective Cohort Study
Source: PLoS Med. 2015 Dec 29;12(12):e1001932. doi: 10.1371/journal.pmed.1001932 (PMC4700973; doi:10.1371/journal.pmed.1001932)
Supplement: S1 Table — (PDF) [file pmed.1001932.s001.pdf]

Table S1: Sensitivity analysis of multivariable model for association between regimen composition and sputum culture conversion, restricted to patients who received no Group 4 or 5 drugs with unknown drug susceptibility (N=440)

		**Adjusted hazard ratio (95% confidence interval)**
Average number of potentially effective drugs received per day		
	0 to <4	**0.48 (0.35–0.65)**
	4 to <5	**0.53 (0.41–0.69)**
	5 to <6	Reference
	6 or more	1.38 (0.95–1.99)
Number of drugs to which resistant at baseline*		1.10 (0.98–1.23)
Resistance pattern at baseline:		
	MDR only	Reference
	MDR with resistance to any second-line injectable	0.63 (0.38–1.06)
	MDR with resistance to any fluoroquinolone	**0.60 (0.38–0.94)**
Previous treatment history		
	None	Reference
	First-line drugs only	0.76 (0.52–1.13)
	Second-line drugs	0.67 (0.43–1.04)
	Unknown	**0.27 (0.08–0.92)**
Smear result		
	Negative	Reference
	Positive	0.60 (0.38–1.06)
	Unknown	**0.27 (0.11–0.71)**
Extent of disease on chest radiograph		
	Unilateral	Reference
	Bilateral	**0.73 (0.56–0.95)**
	Unknown	1.15 (0.49–2.74)

*Continuous variable

Abbreviations: MDR = Multidrug-resistant

Hazard ratios result from multivariable Cox proportional hazard regression model for the association between regimen composition and time to initial sputum culture conversion as the outcome. All variables included in the model are shown, and analysis was stratified by country. The model included the following two interaction terms: average number of effective drugs received per day*average number of untested drugs received per day; average doses of pyrazinamide received per day*average number of untested drugs received per day. Initial sputum culture conversion was defined as at least two consecutive negative cultures of sputum samples collected at least 30 days apart.
