# Supplementary material for: Association between Regimen Composition and Treatment Response in Patients with Multidrug-Resistant Tuberculosis: A Prospective Cohort Study
Source: PLoS Med. 2015 Dec 29;12(12):e1001932. doi: 10.1371/journal.pmed.1001932 (PMC4700973; doi:10.1371/journal.pmed.1001932)
Supplement: S2 Table — (PDF) [file pmed.1001932.s002.pdf]

Table S2: Model 2 adjusted hazard ratio for sputum culture conversion associated with inclusion of one additional effective drug in regimen, stratified by average number of untested drugs received per day

Average number of untested drugs received per day*	Adjusted hazard ratio (95% confidence interval)
0	**1.36 (1.16–1.58)**
1	**1.65 (1.48–1.84)**
2	**2.00 (1.68–2.38)**
3	**2.43 (1.83–3.21)**
4	**2.94 (1.98–4.38)**

*Median: 1; range 0–4

Hazard ratios are adjusted for average number of ineffective drugs received per day, average doses of pyrazinamide received per day, extent and pattern of baseline resistance, previous treatment history, sputum smear result, and extent of disease. Analysis was stratified by country. Initial culture conversion was defined as at least two consecutive negative cultures of sputum samples collected at least 30 days apart.
