# Supplementary material for: Association between Regimen Composition and Treatment Response in Patients with Multidrug-Resistant Tuberculosis: A Prospective Cohort Study
Source: PLoS Med. 2015 Dec 29;12(12):e1001932. doi: 10.1371/journal.pmed.1001932 (PMC4700973; doi:10.1371/journal.pmed.1001932)
Supplement: S1 Text — Checklist of items that should be included in reports of observational studies. (PDF) [file pmed.1001932.s004.pdf]

STROBE Statement—checklist of items that should be included in reports of observational studies

	Item No	Recommendation
**Title and abstract**	1	(*a*) *Indicate the study's design with a commonly used term in the title or the abstract*. The first sentence of the Methods and Findings identifies the study design: "We analyzed data from the Preserving Effective Tuberculosis Treatment Study (PETTS), a prospective observational study of 1,659 adults treated for MDR TB in 9 countries during 2005-2010."
		(*b*) *Provide in the abstract an informative and balanced summary of what was done and what was found*. We summarize in the Methods and Findings section: "We analyzed data from the Preserving Effective Tuberculosis Treatment Study (PETTS), a prospective observational study of 1,659 adults treated for MDR TB in 9 countries during 2005-2010. For all patients, monthly sputum samples were collected, and DST was performed on baseline isolates at the U.S. Centers for Disease Control and Prevention. We included 1,177 patients in our analysis. The primary outcome of interest was initial sputum culture conversion. We used Cox proportional hazards regression, stratifying by country to control for setting-associated confounders, and including clinical and demographic covariates.<br><br>"In multivariable analysis, receiving an average of at least six potentially effective drugs per day (defined as drugs without a DST result indicating resistance) was associated with a 36% greater likelihood of sputum culture conversion than receiving at least five but fewer than six potentially effective drugs (adjusted hazard ratio [aHR] 1.36, 95% confidence interval [CI] 1.09–1.69). Inclusion of pyrazinamide (aHR 2.00, 95% CI 1.65–2.41) or more drugs to which baseline DST indicated susceptibility (aHR 1.65, 95% CI 1.48–1.84 per 1 drug) in regimens was associated with greater increases in the likelihood of sputum culture conversion than including more drugs to which baseline DST indicated resistance (aHR 1.33, 95% CI 1.18–1.51 per 1 drug). Including more drugs for which DST was not performed to the regimen was beneficial only if a minimum of three effective drugs was present in the regimen (aHR 1.39, 95% CI 1.09–1.76 per 1 drug when 3 effective drugs present in regimen)."
**Introduction**		
Background/rationale	2	*Explain the scientific background and rationale for the investigation being reported*. In the first paragraph of the introduction, we describe the current World Health Organization guidelines for constructing MDR TB treatment regimens. In the second paragraph, we summarize recent evidence that suggests that a reassessment of these guidelines may be necessary.
Objectives	3	*State specific objectives, including any prespecified hypotheses*. We summarize the objectives in the last paragraph of the introduction: "To gain insight into how regimen design affects treatment response, we analyzed treatment and microbiological data from the Preserving Effective Tuberculosis Treatment Study (PETTS), a 6-year, multinational, prospective cohort study of patients with MDR TB [6]. We assessed the association between the number of potentially effective drugs included in a regimen and time to sputum culture conversion. In addition, we compared the individual effects of drugs to which DST results indicated susceptibility, drugs to which DST results indicated resistance, and drugs that were not tested."

Methods

Study design	4	*Present key elements of study design early in the paper*. The PETTS study setting, population, and protocol is summarized in the first two paragraph of the methods section (after the ethics statement) and a reference provided to a publication that describes these in detail. This paragraph reads: "The PETTS study design and patient population have been described previously [6]. Briefly, this prospective cohort study, conducted 2005–2010, enrolled consecutive adults with pulmonary MDR TB in nine countries: Estonia (nationwide), Latvia (nationwide), Peru (two districts in Lima), Philippines (greater Manila), Russia (Orel and Vladimir Oblasts), South Africa (Eastern Cape, KwaZulu Natal, Mpumalanga, and Northwest provinces), South Korea (National Masan Tuberculosis Hospital, Masan, and Korean Institute of Tuberculosis, Seoul), Thailand (Sakon Nakon, Srisaket, Ubon Ratchathani, and Yasothon provinces), and Taiwan (nationwide). Inclusion criteria for the study were a positive culture from a specimen collected within 30 days of starting treatment and receipt of second-line drugs for at least 30 days. South Africa restricted enrollment to patients who had not previously been treated for MDR TB. Standardized information was recorded at all sites, including demographic, socioeconomic, and clinical information for each subject, and treatment and laboratory monitoring details. The study was approved by the U.S. Centers for Disease Control and Prevention (CDC) Institutional Review Board (IRB) and IRBs at all participating sites. Written informed consent was obtained from all study participants.<br>Culture was performed on a baseline sputum sample and monthly follow-up sputum samples collected for the duration of treatment. Local laboratories performed cultures for monitoring and DST for determining patient eligibility. A subset of isolates from patients enrolled in the study were shipped in batches to CDC for centralized DST and genotyping."
Setting	5	*Describe the setting, locations, and relevant dates, including periods of recruitment, exposure, follow-up, and data collection*. Study setting, location, and dates are described in the first paragraph of the methods section (after the ethics statement); see text in checklist item 4 above. In addition, a reference is provided for a publication that describes the study setting, location, and dates in greater detail (reference 6).
Participants	6	(*a*) *Cohort study—Give the eligibility criteria, and the sources and methods of selection of participants. Describe methods of follow-up.* The eligibility criteria for the study as a whole are referenced briefly in the methods section (see checklist item 4 above), but described fully in reference 6. The inclusion criteria for this analysis are described fully in the methods section: "Patients were eligible for inclusion in this analysis if they had positive cultures at the start of treatment for MDR TB, if they had DST results from CDC for fluoroquinolones (DST was performed for ciprofloxacin and ofloxacin) and second-line injectable drugs (i.e., amikacin, kanamycin, capreomycin), and if resistance to both isoniazid and rifampin were confirmed at CDC. We excluded patients for whom the DST performed at CDC indicated susceptibility to either isoniazid or rifampin in response to a reviewer suggestion, as several of these patients had been treated with isoniazid or rifampin. Patients with extensively drug-resistant tuberculosis (XDR TB, defined as MDR TB with additional resistance to any fluoroquinolone and at least one second-line injectable drug) were excluded from analysis, as were patients for whom a date of culture conversion or censoring could not be determined."
		(*b*) *Cohort study—For matched studies, give matching criteria and number of exposed*

		and unexposed. Not applicable.
Variables	7	*Clearly define all outcomes, exposures, predictors, potential confounders, and effect modifiers. Give diagnostic criteria, if applicable.* The primary outcome of interest is defined in the first paragraph of the "Definitions" sub-section of the Methods section: "Initial sputum culture conversion was defined as at least two consecutive negative cultures of sputum samples collected at least 30 days apart. Time to sputum culture conversion was defined as the time in days from the start of MDR TB treatment to the sputum specimen collection date of the first of the consecutive negative cultures. Patients for whom sputum culture conversion did not occur were censored 1 month before the collection date of the last sputum specimen because they were still at risk to convert during the last month of follow up."<br><br>The method for calculating and classifying the drug exposures that comprise the primary predictors of interest are summarized in the next two paragraphs.
Data sources/ measurement	8*	*For each variable of interest, give sources of data and details of methods of assessment (measurement). Describe comparability of assessment methods if there is more than one group.* The calculation of the primary predictors of interest (drug-days of exposure) from primary data (days drugs were started and stopped) is described in the last paragraph of the "Definitions" sub-section of the Methods section: "The number of days a drug was included in a patient's regimen was inferred from the days the drug was started and stopped; if a single drug was started and stopped multiple times, the days between each pair of start and stop dates were summed. Drug-days were summed for all the drugs in each of four groups: effective drugs, ineffective drugs, pyrazinamide, and untested drugs. For each group, this sum was divided by the number of days before sputum culture conversion or censoring to calculate the average number of drugs in each group that the patient received per day." In addition, details of all data collection procedures are described in reference 6.
Bias	9	*Describe any efforts to address potential sources of bias.* In the first paragraph of the "Data analysis" sub-section of the Methods section, we state: "We stratified by country to control for setting-associated confounders." This stratification was intended to prevent setting-specific factors from sites that contributed many patients to the cohort from unduly influencing our results.
Study size	10	*Explain how the study size was arrived at.* This is described in great detail in reference 6 (first paragraph of "Statistical analysis" sub-section of Methods section, on page 1410 of publication).
Quantitative variables	11	*Explain how quantitative variables were handled in the analyses. If applicable, describe which groupings were chosen and why.* We describe the grouping of drugs used for analysis of drug exposure (the primary predictor of interest) in the second paragraph of the "Definitions" sub-section of the Methods section: "Classification of each drug's effectiveness was based on results of DST performed at CDC on the baseline culture using the indirect agar plate proportion method [6]. DST was performed for isoniazid, rifampin, ethambutol, ciprofloxacin, ofloxacin, amikacin, capreomycin, kanamycin, streptomycin, rifabutin, ethionamide, and para-aminosalicylic acid. Drugs for which DST indicated susceptibility were considered effective. Drugs for which the baseline DST result indicated resistance were considered ineffective. In addition, levofloxacin and moxifloxacin were considered effective if no resistance to ciprofloxacin or ofloxacin was observed, and were

		considered ineffective if resistance to either ciprofloxacin or ofloxacin was observed. Prothionamide was considered effective if no resistance to ethionamide was observed, and ineffective if resistance to ethionamide was observed. Drugs for which DST was not performed at CDC (cycloserine, terizidone, amoxicillin/clavulanate, clarithromycin, thioacetazone, clofazimine, imipenem, and linezolid) were classified as untested drugs. Pyrazinamide, although not tested routinely, was kept separate from this group of untested drugs because it is a first-line drug with a well-established role in treatment, and it is recommended for routine inclusion in MDR TB regimens [1]."<br><br>We describe the analysis of the primary predictor of interest as both a categorical and continuous variable in the first paragraph of the "Data analysis" sub-section of the Methods section: "…we generated two multivariable models to assess the association between treatment regimen and time to sputum culture conversion. In the first model, the exposure of interest was the average number of potentially effective drugs received per day, analyzed as a categorical variable. In the second model, the exposures of interest were the average number of drugs received in each of the four drug groups, analyzed as continuous variables."
Statistical methods	12	(*a*) *Describe all statistical methods, including those used to control for confounding*. In the first paragraph of the "Data analysis" sub-section of the Methods section, we state: "We analyzed the association between variables of interest and sputum culture conversion using Cox proportional hazards regression. We stratified by country to control for setting-associated confounders. We evaluated proportional hazards assumptions by testing the significance of time-dependent interaction terms for all variables… We considered clinical and demographic covariates for inclusion in the multivariable models based on strength of univariate associations with sputum culture conversion (covariates with Wald P-value <0.1 were eligible for inclusion) or biological plausibility, and we used backward elimination to generate the final models. Resistance pattern at baseline and number of drugs to which the baseline isolate was resistant were retained in both models because of an established association between extent of baseline drug resistance and treatment success [7] and because the extent of drug resistance was likely to be associated with resistance to untested drugs."
		(*b*) *Describe any methods used to examine subgroups and interactions.* In the last paragraph of the Methods section, we state: "In the second model, we believed interactions among the different drug groups to be likely. Therefore, we assessed both the main effects model and a model in which we considered all two-way interaction terms among drug group variables. Collinearity among variables was assessed; a variance inflation factor >5 or a maximum condition index >50 were considered evidence of collinearity."
		(*c*) *Explain how missing data were addressed.* We censored patients who did not experience the primary outcome of interest (sputum culture conversion), but did not exclude them from analysis. We describe the censoring method and rationale in the first paragraph of the "Definitions" sub-section of the Methods section: "Patients for whom sputum culture conversion did not occur were censored 1 month before the collection date of the last sputum specimen because they were still at risk to convert during the last month of follow up."
		(*d*) *Cohort study—If applicable, explain how loss to follow-up was addressed.* See

discussion of censoring in item above.

(e) *Describe any sensitivity analyses.* We tested the robustness of our results by generating two multivariable models using different methods to describe the exposure of interest. In addition, we evaluated the second multivariable model by exploring both a main-effects model and a model incorporating interaction terms. Finally, we carried out a sensitivity analysis in which we excluded patients who received any Group 4 or 5 drug for which susceptibility was unknown. These models are described in the "Data analysis" sub-section of the Methods section: "…we generated two multivariable models to assess the association between treatment regimen and time to sputum culture conversion. In the first model, the exposure of interest was the average number of potentially effective drugs received per day, analyzed as a categorical variable. In the second model, the exposures of interest were the average number of drugs received in each of the four drug groups, analyzed as continuous variables… In the second model, we believed interactions among the different drug groups to be likely. Therefore, we assessed both the main effects model and a model in which we considered all two-way interaction terms among drug group variables.. As a sensitivity analysis, we restricted the first model to patients who did not receive any Group 4 or 5 drugs for which drug sensitivity was unknown."

Continued on next page

**Results**		
Participants	13*	(a) *Report numbers of individuals at each stage of study—eg numbers potentially eligible, examined for eligibility, confirmed eligible, included in the study, completing follow-up, and analysed*. Figure 1 is a flow diagram describing the inclusion and exclusion of patients for this study.
		(b) *Give reasons for non-participation at each stage*. Exclusion reasons are described in Figure 1.
		(c) *Consider use of a flow diagram*. See Figure 1.
Descriptive data	14*	(a) *Give characteristics of study participants (eg demographic, clinical, social) and information on exposures and potential confounders*. These are summarized in Table 1.
		(b) *Indicate number of participants with missing data for each variable of interest*. Unknown values are indicated for each variable where they occur in Table 1.
		(c) *Cohort study—Summarise follow-up time (eg, average and total amount)* In the first paragraph of the results section (page 6), we state: "These patients were followed for a median of 20 months (interquartile range 16–23 months) after MDR TB treatment initiation."
Outcome data	15*	*Cohort study—Report numbers of outcome events or summary measures over time* In the first paragraph of the results section (pages 6-7), we state: "Initial sputum culture conversion occurred for 909 (79,9%) of these patients at a median of 2 months (interquartile range 1–3 months)."
Main results	16	(*a*) *Give unadjusted estimates and, if applicable, confounder-adjusted estimates and their precision (eg, 95% confidence interval).* Make clear which confounders were adjusted for and why they were included. Unadjusted hazard ratios are shown in Table 1 and adjusted hazard ratios in Table 2. All covariates are shown in Table 2. 95% confidence intervals are given in both tables.
		(*b*) *Report category boundaries when continuous variables were categorized*. Exhaustive and non-overlapping categories of numbers of drugs per day are provided in Tables 1 and 2.
		(*c*) *If relevant, consider translating estimates of relative risk into absolute risk for a meaningful time period.* Not applicable.
Other analyses	17	*Report other analyses done—eg analyses of subgroups and interactions, and sensitivity analyses.* Results for both multivariable models are shown in Table 2. An analysis of interactions among 3 drug categories is presented in Table 3. We report no collinearity among variables included in the multivariable models.
**Discussion**		
Key results	18	Summarise key results with reference to study objectives. The first paragraph of the discussion states: "In our analysis, greater numbers of potentially effective drugs in an MDR TB treatment regimen were associated with accelerated sputum culture conversion. In general, inclusion of pyrazinamide or additional drugs to which baseline DST indicated susceptibility (i.e., an effective drug) were associated with greater increases in the likelihood of sputum culture conversion than inclusion of drugs to which baseline DST indicated resistance (i.e., an ineffective drug). The presence of untested drugs in the regimen was associated with an increased likelihood of sputum culture conversion only if a minimum number of effective drugs was present in the regimen."
Limitations	19	*Discuss limitations of the study, taking into account sources of potential bias or imprecision. Discuss both direction and magnitude of any potential bias*. Paragraphs 7-10 of the Discussion articulate numerous limitations of our study, the implication these limitations have on the interpretation of results, and the steps we took to address them (where possible).

Interpretation	20	*Give a cautious overall interpretation of results considering objectives, limitations, multiplicity of analyses, results from similar studies, and other relevant evidence*. We summarize what we believe to be the overall implications of our study in the last paragraph of the manuscript, while pointing out that randomized controlled trials will be needed to definitively demonstrate these conclusions: "In conclusion, our analysis suggests that MDR TB regimens including more potentially effective drugs than the minimum of five currently recommended by WHO may encourage improved response to treatment in patients with MDR TB. In addition, rapid access to high-quality DST results could facilitate the design of more effective individualized regimens. However, randomized controlled trials are necessary to confirm whether individualized regimens with more than five drugs can indeed achieve better cure rates than current recommended regimens, and new rapid DST techniques will be required if knowledge of baseline drug resistance is to guide regimen composition at the beginning of treatment."
Generalisability	21	*Discuss the generalisability (external validity) of the study results*. We summarize other studies that have reported results consistent with our findings throughout the Discussion, but particularly in the second paragraph: "We observed a benefit to receiving a greater number of potentially effective drugs, as well as an interaction in which the presence of more effective drugs enhanced the benefit of untested drugs. Both of these results support existing evidence that increasing the number of drugs in MDR TB regimens is advantageous. Patients receiving individualized regimens containing a minimum of five probably effective drugs for prolonged periods after sputum culture conversion have been shown to have decreased risks of treatment failure, death, and relapse compared to patients who received fewer drugs [3-5]. In addition, high cure rates have been reported with only 9 months of treatment using a standardized regimen including seven drugs during the intensive phase [9]. In response to the accumulating evidence for the benefit of increasing the number of drugs in regimens for MDR TB, WHO guidelines for MDR TB regimen composition increased the minimum number of drugs recommended from four in 2006 and 2008 to five in 2011 [1, 10]. Our results suggest that treatment might be further fortified by adding additional potentially effective drugs."<br><br>In addition, we discuss the limitations and the benefits to using observational data collected within a programmatic context, as was done in this study, in terms of generalizability: "PETTS was an observational study, not a randomized trial. Choice of regimen varied across sites, as not all countries had all drugs available. While we attempted to reduce bias by stratifying analysis by country and including clinical covariates in our analysis, complete elimination of bias in a cohort of this diversity is impossible. Furthermore, the range of baseline resistance patterns and regimens received prevented us from assessing the effects of individual drugs. However, despite the limitations inherent to this type of observational data from treatment programs, conclusions drawn from these data may in fact be more easily translatable to clinical decision-making since they reflect results from ordinary practicePETTS was an observational study, not a randomized trial. In addition, while the study involved sites in nine different countries, our results may not be generalizable to other settings with very different MDR TB epidemics; for instance, the prevalence of pre-XDR fluoroquinolone resistance in the PETTS cohort was substantially lower than has been reported among MDR TB patients in some South Asian settings [19, 20]. Choice of regimen varied across sites, as not all countries had all drugs available. While we attempted to reduce bias by stratifying analysis by country and including clinical covariates in our analysis, complete elimination of bias in a cohort of this diversity is impossible. However, despite the limitations inherent to this type of observational data from treatment programs, conclusions drawn from these data may in fact be more easily

		translatable to clinical decision-making since they reflect results from ordinary practice.”
**Other information**		
Funding	22	*Give the source of funding and the role of the funders for the present study and, if applicable, for the original study on which the present article is based. The funding statement indicates the funders of the original study on which this manuscript is based; no additional funding was required to produce this manuscript.* “This work was supported by the U.S. Agency for International Development, U.S. CDC, U.S. National Institutes of Health’s Division of Intramural Research of the National Institute for Allergy and Infectious Diseases, and the Korean Ministry of Health and Welfare. CDC Division of Tuberculosis Elimination led the study design, training for data collection and monitoring, data analysis, data interpretation, and writing of the report. Other sponsors had no roles in these activities. The views and opinions expressed in this article are those of the authors and do not necessarily represent an official position of the U.S. Centers for Disease Control and Prevention.”

*Give information separately for cases and controls in case-control studies and, if applicable, for exposed and unexposed groups in cohort and cross-sectional studies.

Note: An Explanation and Elaboration article discusses each checklist item and gives methodological background and published examples of transparent reporting. The STROBE checklist is best used in conjunction with this article (freely available on the Web sites of PLoS Medicine at http://www.plosmedicine.org/, Annals of Internal Medicine at http://www.annals.org/, and Epidemiology at http://www.epidem.com/). Information on the STROBE Initiative is available at www.strobe-statement.org.
