# Supplementary material for: Association between Regimen Composition and Treatment Response in Patients with Multidrug-Resistant Tuberculosis: A Prospective Cohort Study
Source: PLoS Med. 2015 Dec 29;12(12):e1001932. doi: 10.1371/journal.pmed.1001932 (PMC4700973; doi:10.1371/journal.pmed.1001932)
Supplement: S2 Text — Original plan for primary analysis that was submitted as part of the institutional-review-board-approved PETTS protocol. (PDF) [file pmed.1001932.s005.pdf]

Data Analysis Plan

Using clinical, laboratory, and programmatic data, general descriptive statistics will be done, incidence estimates derived, and predictors for amplification will be identified and examined. Drug susceptibility test results from different test methods will be compared with each other and with the nucleotide sequence of mycobacterial DNA. Univariate, bivariate, multivariate analysis, and survival analysis will be done using a statistical computer program such as SAS 9.0. Differences among non-GLC sites will be considered when being compared to GLC sites. Programmatic information such as the degree of incorporation of the 5 principles of the WHO DOTS strategy for TB control [13] will be used to examine for variation between non-GLC sites. The details of analysis in regards to each of the study objectives is as follows.

The prevention of amplification will be compared between GLC and non-GLC sites by first comparing the incidence of amplification at GLC sites versus non-GLC sites. Bivariate analysis will also be performed as shown in the 2x2 table below:

	Amplification	
	Yes	No
GLC Site		
Non-GLC Site		

Because we will have time to amplification of resistance to anti-TB drugs if it occurs, we will perform a survival analysis to examine differences in demographic and clinical variables between GLC versus non-GLC sites. Proportionality will be checked for these variables of interest by looking for a constant (stable proportionate) difference between plots of estimates of the log(e) cumulative hazard. Based on these tests of proportionality, parametric or semiparametric modelling will be performed.

To compare accessibility to second-line TB drugs at GLC versus non-GLC site, univariate analysis will be used to examine and describe programmatic information. More specifically, the factors to be examined include where drugs are acquired, the presence or absence of drug shortage, and any delay or lack of delay in acquisition of drugs due to the GLC process. At GLC sites we will look at the number of patients treated with GLC drugs versus a combination of GLC and non-GLC drugs as well as the number of patients that could have been treated without the GLC discount. For non-GLC sites access to treatment will be evaluated by examining the length of time to drug procurement. For both GLC and non GLC sites, we will also compare measures for drug quality control.

The risk factors for amplification of resistance will be examined using bivariate and multivariate analysis. The outcome variable of interest is the presence or absence of amplification. The principle independent variable of interest is GLC versus non-GLC site. Because there are other risk factors that may affect amplification and confound the relationship between amplification and site(GLC vs non-GLC), other possible risk factors for resistance amplification will be examined including history of TB treatment default, other interruptions in treatment, poor nutritional status, and alcohol use. These other risk factors will also be examined at GLC sites and non-GLC sites separately as shown in the 2x2 tables below. Multivariate analysis will also be used to examine these risk factors. . For multivariate models of risk factors for amplification of resistance, we will include relevant variables with p values <0.26

from bivariate analysis. Correlation between variables to be included in multivariate analysis will be checked.

	Amplification: Yes	Amplification: No
GLC Site		
Non-GLC Site		

		Amplification: Yes	Amplification: No
Default	Yes		
	No		

Non-GLC Site:

		Amplification: Yes	Amplification: No
Poor nutrition	Yes		
	No		

Non-GLC Site:

		Amplification: Yes	Amplification: No
Alcohol abuse	Yes		
	No		

Success rates of MDR TB treatment will be determined at GLC versus non-GLC sites and compared. The outcome variable of interest will be treatment success and the independent variables to be examined will be site (GLC versus non-GLC) and presence of amplification.

Overall

		Treatment Success: Yes	Treatment Success: No
Amplification	Yes		
	No		

Overall

	Treatment Success: Yes	Treatment Success: No
GLC Site		
Non-GLC Site		

GLC Site:

		Treatment Success: Yes	Treatment Success: No
Amplification	Yes		
	No		

Non- GLC Site:

		Treatment Success: Yes	Treatment Success: No
Amplification	Yes		
	No		
