# Supplementary material for: Association between Regimen Composition and Treatment Response in Patients with Multidrug-Resistant Tuberculosis: A Prospective Cohort Study
Source: PLoS Med. 2015 Dec 29;12(12):e1001932. doi: 10.1371/journal.pmed.1001932 (PMC4700973; doi:10.1371/journal.pmed.1001932)
Supplement: S3 Text — Earliest articulation of planned secondary analyses; secondary analyses were understood by all investigators to be dynamic and not limited to those identified in this presentation. (PDF) [file pmed.1001932.s006.pdf]

PETTS Data Analysis Plan

Kurbatova Ekaterina

PETTS Global Investigators Meeting
Berlin, Germany
16 November 2010

Analysis, Publication of Results

- **Framework with 3 levels:**
  - Primary: 4 reports focusing on main objectives of PETTS
  - Secondary: Multiple other analyses & publications initiated by partners
  - Site-specific analyses, publications & presentations (oral, posters, abstracts)

PRIMARY ANALYSES AND REPORTS

Primary Analyses and Reports

- **#1 - Prevalence of and risk factors for baseline drug resistance**
  - To determine baseline prevalence of and clinical, epidemiological, and microbiological risk factors for resistance to second-line drugs (SLD)
- **Comments**
  - Manuscript in CDC clearance!
- **Point of contact (POC)***
  - Tracy Dalton, Peter Cegielski

*POC will work with the coordinating center team to finalize data, and will work with coordinating team and country representatives on data analysis and report writing

Primary Analyses and Reports

- **#2 - Incidence of and risk factors for acquired drug resistance**
  - To determine the incidence of and risk factors for amplification of drug resistance
- **Comments**
  - Per protocol, genotyping was to be based on comparison of DST results of all drugs
  - SM, EMB, PZA, THA, PAS – poor reproducibility of DST results
  - Should genotyping isolate-pairs with differing DST results be limited to INH, RIF, FQ, SL-INJ?
- **Point of contact (POC)**
  - Peter Cegielski, Tracy Dalton

Primary Analyses and Reports

- **#3 - Effect of acquired drug resistance on treatment outcomes**
  - To describe MDR-TB treatment outcomes and to determine risk factors for poor treatment outcomes
  - To evaluate time to and identify independent predictors of time to sputum culture conversion
  - To assess impact of acquired drug resistance and delayed sputum culture conversion on treatment outcome
- **Point of contact**
  - Peter Cegielski, Tracy Dalton, Ekaterina Kurbatova, Julia Ershova

Primary Analyses and Reports

- **#4 - Timing of acquired drug resistance in relation to specific mutations**
  - To determine time to change in drug resistance and risk factors for faster amplification of resistance
  - To assess if specific mutations are associated with time to resistance amplification
- **Comments**
  - DST and genotyping for those with different baseline & final DST (INH, RIF, FQ, SL-INJ) will be done selectively according to an algorithm
- **Point of contact**
  - Tracy Dalton, Peter Cegielski, Ekaterina Kurbatova, Julia Ershova

SECONDARY ANALYSES AND REPORTS

Secondary Analyses and Reports

- **HIV infection: acquired resistance and treatment outcomes in relation to CD4 count and antiretroviral therapy**
  - To better characterize the subset of HIV-positive patients with MDR-TB
  - To assess if HIV infection is a specific risk factor for acquired/amplified resistance to SLD, and longer time to culture conversion, compared to HIV negative patients
  - To assess impact of use of antiretroviral (ARV) therapy and co-trimoxazole preventive treatment (CPT) on mortality among HIV-infected patients
- **Point of contact**
  - Charlotte Kvasnovsky, Ekaterina Kurbatova, Melanie Wolfgang, Julia Ershova

Secondary Analyses and Reports of Aggregate Data

- **Genotyping differences in sequential isolates of *Mycobacterium tuberculosis* from same individuals**
  - To describe rates and probable reasons for changed genotyping profiles (exogenous re-infection, lab cross-contamination, mixed infection).
- **MDR-TB with 2 or more strains**
  - Reasons for change in genotypes between baseline and final isolates, e.g., reinfection, mixed infection, cross-contamination
  - Impact on treatment outcomes
- **Point of contact**
  - Ekaterina Kurbatova, Peter Cegielski

Secondary Analyses and Reports

- **Association between molecular genetic basis of drug resistance and treatment outcome**
  - To determine whether there is an association between specific genetic mutations that confer drug resistance (GenoType MTBDR*plus,* HAIN Lifescience) and clinical outcome, specifically the outcomes of development of additional drug resistances and duration of persistent positive culture
- **Point of contact**
  - Eleanor Click, Heather Alexander, Tracy Dalton

Secondary Analyses and Reports

- **Evaluation of GenoType MTBDR*plus* and MTBDR*sl* (HAIN Lifescience)**
  - To determine the accuracy of the *MTBDRplus* assay (HAIN Lifescience) for detection of Rif-resistance, INH-resistance and MDR TB in clinical isolates
  - To evaluate the use of MTBDR*plus* for differentiating between high- and low-level INH resistance
  - To determine the accuracy of the MTBDR*sl* assay for detection of resistance to injectable SLD, the fluoroquinolones, and EMB
- **Point of contact**
  - Heather Alexander, Tracy Dalton

Secondary Analyses and Reports

- **Time-to-diagnosis of drug resistance and time-to-initiation of appropriate treatment: impact on acquired resistance and outcomes**
  - To determine if longer time to diagnosis and treatment of MDR-TB was associated with baseline drug resistance and poor treatment outcomes
- **Point of contact**
  - Peter Cegielski, Tracy Dalton, Ekaterina Kurbatova

Secondary Analyses and Reports

- **Impact of hospitalization on development of additional drug resistance due to re-infection with a different strain**
  - To determine effect of hospitalization on rates of drug resistance (baseline and acquired) because of re-infection, and association with nosocomial transmission
- **Point of contact**
  - Ekaterina Kurbatova, Peter Cegielski, Julia Ershova

Secondary Analyses and Reports

- **Application of Molecular Detection of Drug Resistance (MDDR) service to PETTS isolates: rapid predictors of XDR-TB**
  - To use DNA sequencing to further elucidate associations between genotypic and phenotypic SLD resistance in *M. tuberculosis* and clinical outcome in MDR-TB patients
  - To gain a better understanding of the extent of cross-resistance within drug classes and to further characterize mutations associated with cross-resistance
  - To identify potential novel mechanisms of resistance to the injectable SLDs (KAN, AMK, and CAP) and the fluoroquinolones (CIP, OFL, and MOX)
- **Point of contact**
  - Tracy Dalton

Secondary Analyses and Reports

- **Baseline and acquired resistance to pyrazinamide (PZA) among MDR-TB patients - prevalence and effect on treatment outcomes**
  - To describe rates and predictors of baseline and acquired resistance to PZA and effect on treatment outcomes
- **Comments**
  - Should we test for PZA resistance using a different method?
  - Should we select isolates for PZA testing according to some algorithm or decision rule?
- **Point of contact**
  - Peter Cegielski, Tracy Dalton, Lois Diem, Beverly Metchock, James Posey, Bonnie Plikaytis, Michael Iademarco, Ekaterina Kurbatova, Andrew Vernon

Secondary Analyses and Reports of Aggregate Data

- **Diabetes mellitus and MDR-TB outcomes**
  - To asses if diabetes is a specific risk factor for longer time to culture conversion, and for baseline and acquired/amplified resistance to SLDs
  - To compare treatment outcomes between patients with/without diabetes.
- **Impact of body-mass index on treatment outcomes of patients with MDR-TB**
  - To determine the impact of low BMI on acquired resistance and treatment outcome.
